# Treatment Sequencing and Independent Outcomes of First- and Second-Line Chemotherapy in a Retrospective Series of Patients with Biliary Tract Cancer

**DOI:** 10.3390/jcm13237262

**Published:** 2024-11-29

**Authors:** Giorgio Frega, Andrea Palloni, Chiara Deiana, Alessandro Rizzo, Angela Dalia Ricci, Giovanni Brandi

**Affiliations:** 1 Osteoncology, Soft Tissue and Bone Sarcomas, Innovative Therapy Unit, IRCCS Istituto Ortopedico Rizzoli, 40136 Bologna, Italy; giorgio.frega@ior.it; 2 Medical Oncology, IRCCS Azienda Ospedaliero-Universitaria di Bologna, 40138 Bologna, Italy; andrea.palloni@aosp.bo.it (A.P.); chiarad.kw@gmail.com (C.D.); 3 Struttura Semplice Dipartimentale di Oncologia Medica per la Presa in Carico Globale del Paziente Oncologico “Don Tonino Bello”, IRCCS Istituto Tumori “Giovanni Paolo II”, Viale Orazio Flacco 65, 70124 Bari, Italy; rizzo.alessandro179@gmail.com; 4 Medical Oncology Unit, National Institute of Gastroenterology, “Saverio de Bellis” Research Hospital, 70013 Castellana Grotte, Italy; dalia.ricci@gmail.com

**Keywords:** biliary tract cancer, cholangiocarcinoma, sequential treatment, chemotherapy, first line, second line

## Abstract

**Background/Objectives:** Biliary tract cancers (BTCs) are aggressive neoplasms with limited therapeutic options. The amount of prospective evidence is poor, and limited data are available on the impact of treatment sequencing on survival. Here we report a real-world experience of patients with advanced BTC treated with at least three lines of therapy. We evaluated the impact of sequential treatments, and we further compared the efficacy of Gemcitabine/Cisplatin (GemCis) and mFOLFOX to other first- and second-line chemotherapy regimens, respectively. **Methods**: Data on 60 patients with locally advanced or metastatic BTC under the care of a single Italian referral hospital and treated with at least three lines of chemotherapy were retrospectively collected. Data from 56 patients were included in the analysis. Survival analyses were performed using R software (v1.2.5042). **Results**: We compared the outcomes of patients treated according to the “standard” pre-immunotherapy sequence (GemCis and mFOLFOX in the first and second lines, respectively) versus those treated with all other combinations (“control” group). Our analysis did not show significant survival differences between the two groups. However, it should be noted that we selected long-survival patients by including only those who received at least three or more lines of chemotherapy. Focusing on the first-line setting, no significant differences in both mPFS and mOS emerged by comparing GemCis versus other doublets (mainly Gemcitabine/Oxaliplatin). Similarly, mPFS and mOS from second-line treatment did not statistically differ between patients treated with mFOLFOX versus those treated with other regimens (71% chemotherapy doublets). **Conclusions:** Our series provides real-world outcomes of patients with advanced BTC before the approval of immunotherapy. Even considering the monocentric and retrospective design, our study represents one of the first analyses on the impact of sequential treatment strategies in patients with BTC.

## 1. Introduction

Biliary tract cancers (BTCs) are aggressive neoplasms with a significant worldwide variation of incidence rates and with limited therapeutic options [1,2,3]. The number of prospective available trials is limited. Surgery represents the only potentially curative option, but it is feasible only in a small percentage of cases, and even in patients who can undergo surgical resection, the recurrence rate is high [4,5].

For more than a decade, GemCis has been the standard first-line treatment option for patients with advanced BTC (ABC-02 trial). [6] Recently, the addition of immunotherapy to GemCis significantly improved median overall survival (mOS) in advanced patients who are chemo-naïve [7,8]. In the second-line setting, mFOLFOX showed a small but significant benefit compared to active symptom control (ABC-06 trial) [9]. Innovative drugs, designed to target specific molecular pathways, were proposed and evaluated for selected subgroups of patients [10,11,12,13]. In the last years, encouraging data led to the approval of targeted therapies in subgroups of BTCs with specific molecular alterations, such as FGFR2 rearrangements and IDH-1 mutations [12,13]. The role of radiotherapy remains still limited in both the adjuvant and advanced settings, but the recent advent of immunotherapy could encourage a potential combined approach [14,15]. To date, chemotherapy currently remains the backbone of the available therapeutic arsenal for the majority of patients. To date, few studies have focused on the impact of different treatment sequencing.

In our paper, we propose an exploratory investigation of the relevance of treatment sequencing in heavily treated patients for advanced disease. We retrospectively evaluated the outcome of patients treated with chemotherapy strategies available in clinical practice before immunotherapy approval by comparing GemCis with other chemotherapy doublets (mainly GemOx) in the first-line setting as well as mFOLFOX with other combinations in the second-line setting.

## 2. Methods

**Figure 1:**
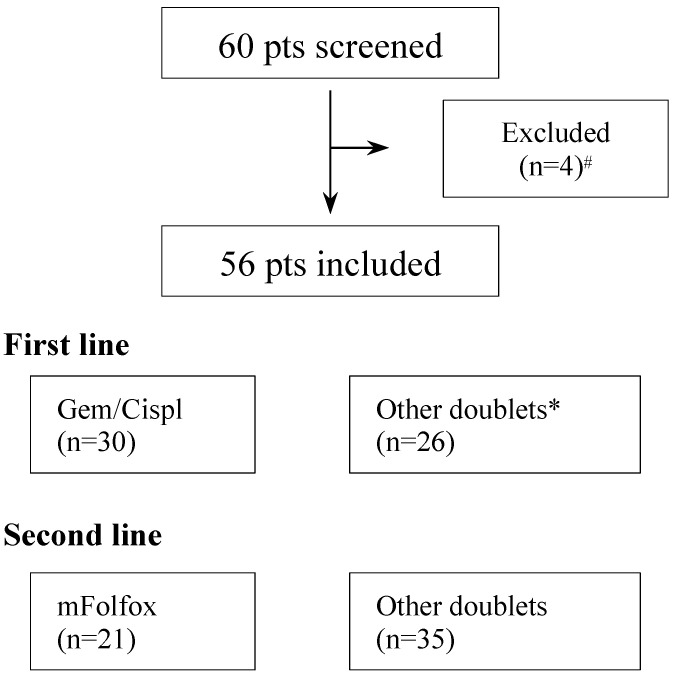
Flowchart of the study. ^#^ One patient treated with first-line Atezolizumab/Bevacizumab combination and three patients treated with first line single agent chemotherapy. * A total of 35 out of 36 patients treated with GemOx combination.

We conducted a retrospective study including patients with intrahepatic/extrahepatic cholangiocarcinoma and gallbladder cancer treated for advanced disease in our referral hospital (IRCCS Azienda Ospedaliero-Universitaria di Bologna) from 2005 to 2022. We included patients aged 18 years or older with histologically confirmed and unresectable/advanced disease who were treated with a first-line chemotherapy combination (GemCis or other doublets) and received at least three lines of chemotherapy (CTX). Data from 60 patients were retrospectively collected. Fifty-six patients were included in the analysis. Four patients were not included due to the following reasons: one patient was treated with first-line Atezolizumab/bevacizumab combination, and three patients were treated with first-line single-agent CTX (Figure 1).

For survival analyses, we evaluated the median overall survival (mOS), defined as the time interval from the first CTX cycle (of the first- or second-line regimen, respectively) to the last follow-up visit or death from any cause. We also evaluated the progression-free survival (PFS) for the first-line and second-line treatment (defined as the time between the first cycle of CTX and disease progression or death from any cause). Patients were treated according to the ethical guidelines of the 1975 Declaration of Helsinki. The study was conducted under the protocol CE 228/2017/O/Oss (approved by the Area Vasta Emilia Centro Ethics Committee).

The flowchart of the study is shown in Figure 1.

The statistical analysis was performed by using the Kaplan–Meier estimator and the log-rank test to make comparisons between curves. R statistic was employed as the main statistical software (v1.2.5042).

## 3. Results

The median age of the patients included (n = 56) was 61 years (range 29–79 years). The majority of patients had intrahepatic cholangiocarcinoma (n = 40; 71.4%). All patients had unresectable advanced disease and were treated with at least three lines of chemotherapy.

The baseline characteristics of patients included are reported in Table 1, summarized according to the first- and second-line CTX regimens administered. The mOS of the entire series of patients was 20.3 months (95% CI: 16.8–23.9).

### 3.1. mOS According to Treatment Sequencing

**Figure 2:**
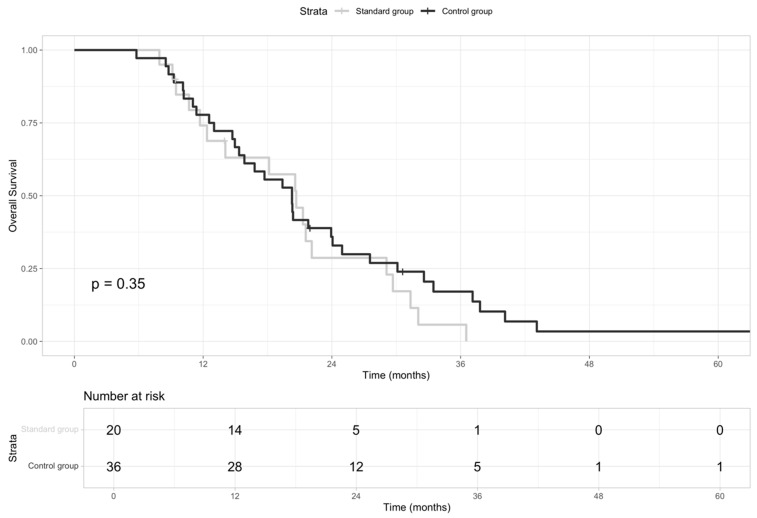
Kaplan–Meier curves of OS for patients who received the standard sequential treatment (“Standard group”) vs. those who received any other sequential therapies (“Control group”).

Firstly, we compared the outcome of patients (n = 20) who received the standard (“standard group”) sequential treatment strategy before immunotherapy approval (GemCis as first-line and mFOLFOX as second-line) versus those (n = 36) who received any other sequential therapies (“control group”). We did not observe any statistical differences in mOS (HR: 0.75, 95% CI: 0.42–1.36; *p*-value 0.35) (Figure 2).

### 3.2. mOS and mPFS According to First-Line Treatment

**Figure 3:**
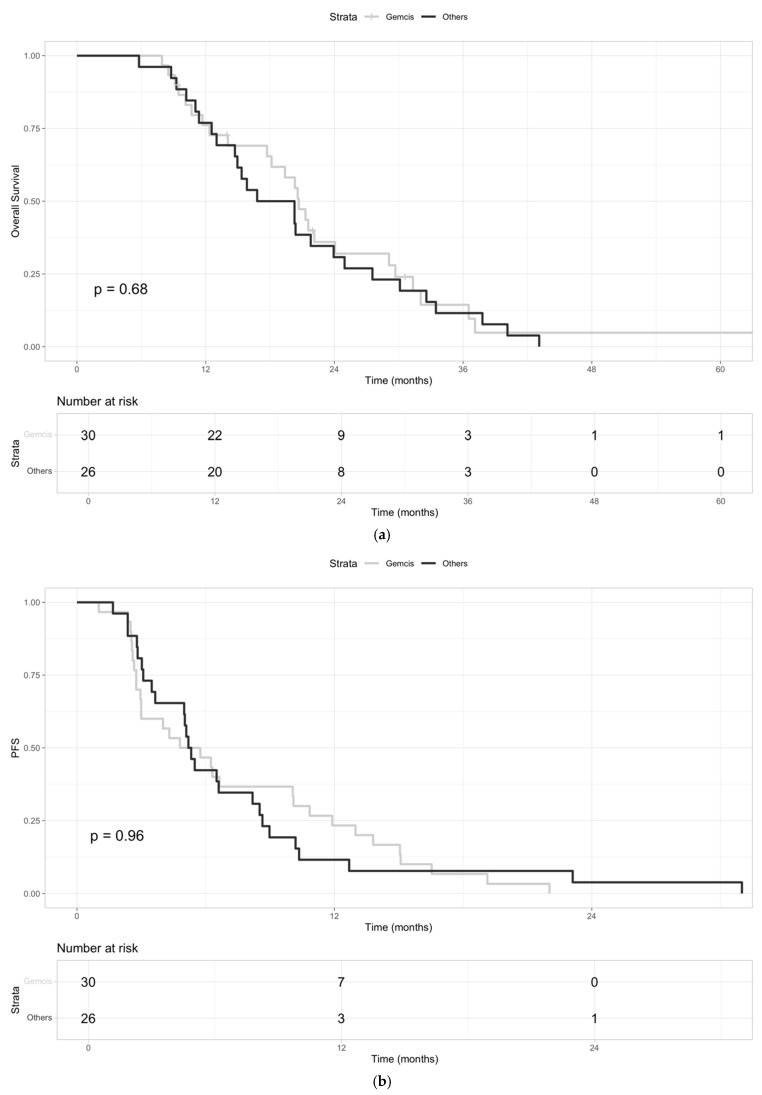
(**a**) Kaplan–Meier curves of OS for patients who received GemCis (“Gemcis”) versus those treated with any other doublet chemotherapies as first-line treatment (“Others”). (**b**) Kaplan–Meier curves of PFS for patients who received GemCis (“Gemcis”) versus those treated with any other doublet chemotherapies as first-line treatment (“Others”).

We then evaluated the mOS of the patients according to which the first line of chemotherapy was administered. No statistical differences emerged in mOS between patients who received GemCis (n = 30) and those treated with any other CTX doublets (n = 26). The mOS was 20.7 months (95% CI: 18.5–29.7) and 18.5 months (95% CI: 14.7–27.5), respectively (HR: 1.12, 95% CI: 0.65–1.95; *p*-value = 0.68) (Figure 3a).

The PFS was 5.3 (95% CI: 3.0–10.8) and 5.3 (95% CI: 3.6–8.6) months for GemCis and for other first-line treatments, respectively (HR: 1.01, 95% CI: 0.59–1.75; *p*-value = 0.96) (Figure 3b).

Of note, 25 out of 26 patients (96%) in the control group received the Gemcitabine/Oxaliplatin (GemOx) combination, while only one patient received mFOLFOX as the first-line treatment.

### 3.3. mOS and mPFS According to Second-Line Treatment

**Figure 4:**
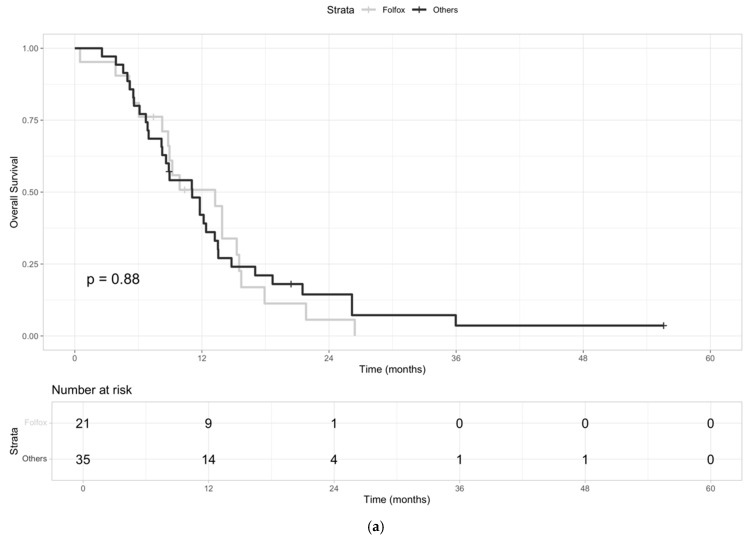

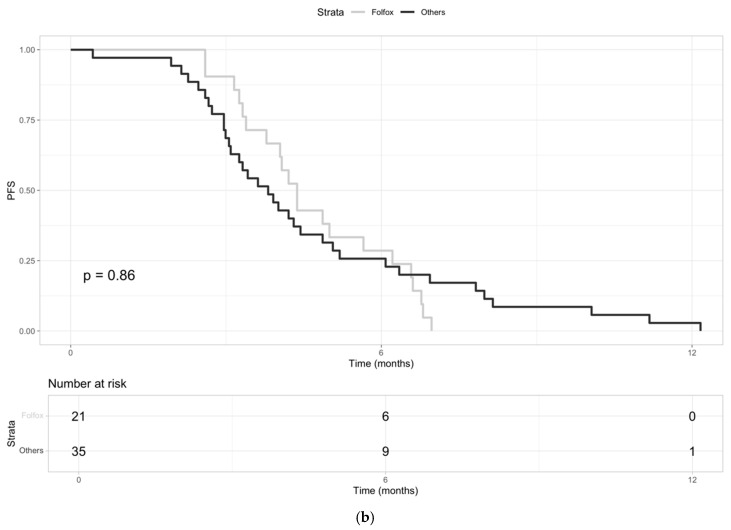
(**a**) KM curves of OS from the second line treatment for patients who received mFOLFOX (“Folfox”) versus those who received any other therapies (“Others”). (**b**) KM curves of PFS from the second line treatment for patients who received mFOLFOX (“Folfox”) versus those who received any other therapies (“Others”).

We further estimated the mOS from the second-line treatment in patients who received mFOLFOX (n = 21) versus those who received any other therapies (n = 35). The chemotherapy regimens administered are reported in Table 1. More in detail, 65.7% (23/35) of the patients received a CTX doublet (gemcitabine/capecitabine, mFOLFIRI, mFOLFOX, carboplatin/gemcitabine), 22.9% (8/35) a CTX single agent (gemcitabine or capecitabine), and 11.4% (4/35) TKIs or mAbs (TKI anti-FGFR, sorafenib, mAb anti-HER2). The mOS from the start of second-line therapy was 13.2 months (95% CI: 8.8–15.7) and 11.1 months (95% CI: 8.3–13.5) for mFOLFOX and other second-line doublets, respectively (HR 0.96, 95% CI: 0.54–1.70; *p*-value = 0.88) (Figure 4a). The mPFS was 4.4 months (95% CI: 3.8–6.6) and 3.8 months (95% CI: 3.1–5.1) for mFOLFOX and other second-line doublets, respectively (HR 0.71, 95% CI: 0.39–1.28; *p*-value = 0.86) (Figure 4b).

We further compared the second-line mOS and mPFS of patients treated with mFOLFOX (n = 21) versus those treated with mFOLFIRI (n = 6). No significant differences emerged in either mOS or mPFS: HR: 2.11 (95% CI: 0.70–6.32; *p*-value = 0.18) and HR: 1.31 (95% CI: 0.47–3.65; *p*-value = 0.6), respectively (Supplementary Figure S1a/b).

## 4. Discussion

Until recently, the GemCis combination was considered the best first-line therapeutic option for patients with advanced cholangiocarcinoma following the results of the ABC-02 and BT22 trials [6,16]. In 2021, a further randomized clinical trial showed a small but significant benefit from mFOLFOX over active symptom control as second-line treatment [9].

Here we report a retrospective series of long-term surviving patients with BTC treated with at least three lines of chemotherapy for advanced disease in the pre-immunotherapy era.

All patients were treated with a chemotherapy doublet in the first line, and over 70% received a combination regimen in the second line as well.

In our analysis, we did not observe any statistically significant differences in mOS between patients treated with GemCis and then mFOLFOX versus those who received other treatment sequences. This result is probably influenced by the decision to include only patients who received at least three or more lines of treatment, thus selecting patients with a better prognosis or, conversely, those with rapid disease progression but preserved clinical conditions. Furthermore, nearly all patients received gemcitabine, a platinum derivative, and a fluoropyrimidine at some point during their medical history.

Regarding the first-line setting, unlike the ABC-02 trial, in which the control arm was gemcitabine monotherapy, we compared the mOS of patients treated with GemCis with those who received other doublet CTX regimens (with 96% of the latter group receiving GemOx). The lack of statistically significant differences in mOS and mPFS between these first-line doublets may be indicative of a good response of BTCs to all platinum-based doublets. We acknowledge that patients treated in first-line with oxaliplatin (instead of cisplatin) were treated before the ABC-02 trial or presented clinical contraindications to cisplatin administration, possibly influencing their prognosis.

Moving on to second-line treatment, we did not identify a difference in mPFS or mOS for patients treated with mFOLFOX versus the “other therapies” group, nor versus mFOLFIRI in a head-to-head comparison. It should be noted that the majority of patients included in the “other therapy” group received a chemotherapy doublet (71%). However, this negative result should be weighted considering that in the ABC 06 trial the control arm was active symptom control [9,17].

Furthermore, a previous retrospective study did not show significant survival differences between fluoropyrimidine monotherapy and doublet regimens in the second-line setting [18], and a Korean randomized phase 2 study on 118 patients with BTC pre-treated with first-line GemCis did not show any statistical differences between modified FOLFOX and modified FOLFIRI in terms of response rate, PFS (2.8 vs. 2.1 months, respectively), and OS (6.3 vs. 5.7 months, respectively). A recent randomized phase 2b trial (NIFTY trial) demonstrated that adding liposomal irinotecan to treatment with FU/LV significantly improved mPFS in patients with advanced BTC progressing to GemCis [19].

Undoubtedly, the major limitation of our analysis is the retrospective design and related biases. In this specific exploratory analysis, we included only patients who received at least three lines of treatment, and given the small number of patients, our study did not reach statistical significance. However, in our analysis we did not investigate the impact of baseline patient characteristics (e.g., ECOG PS, primary tumor location, disease status, biliary drainage, serum tumor markers, etc.).

We also acknowledge that the mOS of our series, both from first-line and second-line treatment, are longer than those reported in prospective randomized pivotal trials (ABC-02 and ABC-06), probably because we selected long-term surviving patients [6,9].

In conclusion, these data could shed light on the need to define an optimal sequential strategy in patient management and encourage the identification of an optimal backbone sequence for subgroups of patients with advanced BTCs, particularly with the advent of new treatment options, including chemo-immunotherapy combinations and targeted agents.

More robust evidence is needed on the impact of treatment sequencing, such as through analysis of shared multicenter registries.

## Figures and Tables

**Table 1:** Treatment sequencing. Baseline characteristics of patients treated with “standard” sequential treatment (“Standard group”) vs. those who received any other sequential therapies chemotherapy (“Control group”).

**Standard group** First line: Gemcitabine/Cisplatin Second line: mFOLFOX	n 20 (35.7%)
Age	Median 60 years (42–74)
Sex	Male = 11 (55%)
	Female = 9 (45%)
**Control group** All other treatment sequencing in first line and second line	n 36 (64.3%)
Age	Median 62 years (29–78)
Sex	Male = 19 (52.8%)
	Female = 17 (47.2%)
**First line**	
Gemcitabine/Cisplatin	n 10 (27.8%)
Gemcitabine/Oxaliplatin	n 25 (69.4%)
mFolfox	n 1 (2.8%)
**Second line**	
Gemcitabine/Capecitabine	n 16 (44.4%)
mFOLFIRI	n 6 (16.7%)
Gemcitabine	n 6 (16.7%)
Capecitabine	n 2 (5.6%)
TKI anti-FGFR	n 2 (5.6%)
Sorafenib	n 1 (2.8%)
mAb anti-HER2	n 1 (2.8%)
Carboplatin/Gemcitabine	n 1 (2.8%)
mFOLFOX	n 1 (2.8%)

## Data Availability

The data that support the findings of this study are available upon request from the corresponding author.

## Supplementary Materials

The following supporting information can be downloaded at: https://www.mdpi.com/article/10.3390/jcm13237262/s1, Figure S1: (a) KM curves of OS from the second line treatment for patients who received mFOLFOX versus those treated with mFOLFIRI. (b) KM curves of PFS from the second line treatment for patients who received mFOLFOX versus those treated with mFOLFIRI.

## Abbreviations

BTC= biliary tract cancers
CTX = chemotherapy
GBC = gallbladder cancer
GemCis = Gemcitabine/Cisplatin
GemOx = Gemcitabine/Oxaliplatin
iCCA = intrahepatic cholagiocarcinoma
pCCA = perihilar cholagiocarcinoma
dCCA = distal cholagiocarcinoma
KM = Kaplan–Meier
mOS = median overall survival
mPFS = median progression-free survival
mo = months
RT = radiotherapy
vs = versus
