# Supplementary material for: Treatment Sequencing and Independent Outcomes of First- and Second-Line Chemotherapy in a Retrospective Series of Patients with Biliary Tract Cancer
Source: J Clin Med. 2024 Nov 29;13(23):7262. doi: 10.3390/jcm13237262 (PMC11642358; doi:10.3390/jcm13237262)
Supplement: Supplementary file 1 [file jcm-13-07262-s001.zip › jcm-3192533-supplementary.pdf]

Supplementary Materials

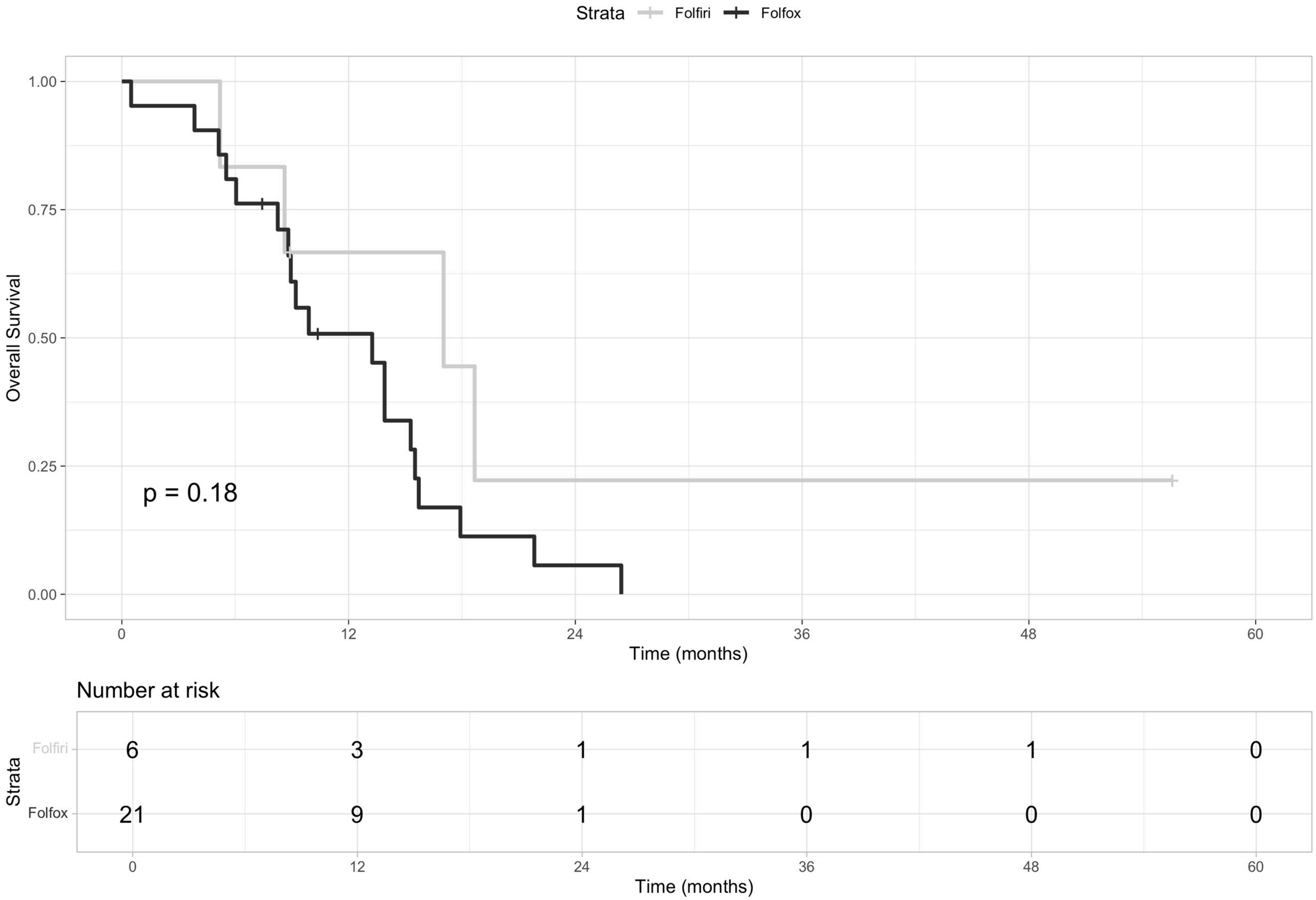

(**a**)

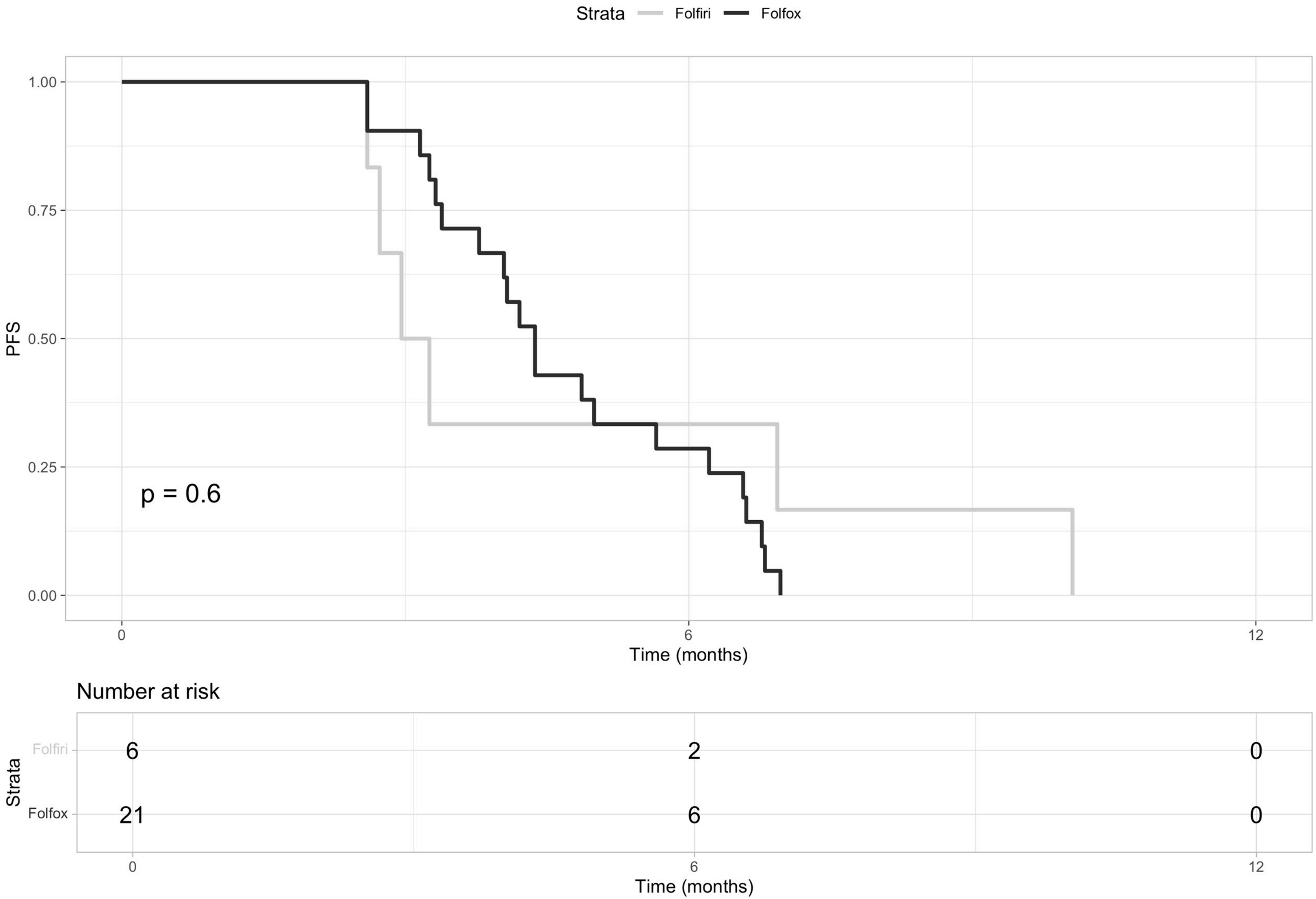

(**b**)

Figure S1. (**a**) KM curves of OS from the second line treatment for patients who received mFOLFOX versus those treated with mFOLFIRI. (**b**) KM curves of PFS from the second line treatment for patients who received mFOLFOX versus those treated with mFOLFIRI.
